# The pathogenic role of double-negative B cells in autoimmune diseases

**DOI:** 10.3389/fimmu.2026.1839432

**Published:** 2026-07-27

**Authors:** Yanzuo Wu, Ze Yang, Shuo Huang, Yongsheng Fan, Guanqun Xie

**Affiliations:** 1 The First School of Clinical Medicine, Zhejiang Chinese Medical University, Hangzhou, Zhejiang, China; 2 School of Basic Medical Sciences, Zhejiang Chinese Medical University, Hangzhou, Zhejiang, China; 3 Department of Rheumatology, The Second Affiliated Hospital of Zhejiang Chinese Medical University, Hangzhou, Zhejiang, China

**Keywords:** autoimmune diseases, double-negative B cells, extra-follicular pathway, rheumatoid arthritis, systemic lupus erythematosus

## Abstract

Double-negative (DN) B cells have emerged as pivotal players in autoimmune diseases, transitioning from an overlooked exhausted subpopulation to a lineage with potent pathogenic potential. Based on surface marker expression, DN B cells are subdivided into four distinct subsets: DN1, DN2, DN3, and DN4. This review provides a comprehensive overview of these subsets, focusing on their origins, phenotypes, and developmental trajectories. In disorders such as systemic lupus erythematosus (SLE), rheumatoid arthritis (RA), primary Sjögren**’**s syndrome (pSS), and multiple sclerosis (MS), expanded DN B cells infiltrate target organs via chemokine receptor-mediated pathways and interact with T cells to drive the pathological remodeling of the local immune microenvironment. Elucidating the regulatory mechanisms of DN B cell differentiation and their functional heterogeneity across disease contexts will provide a critical foundation for the development of targeted precision immunotherapies.

## 1 Introduction

The incidence of autoimmune diseases is exhibiting a year-on-year upward trend, establishing them as the third-largest chronic disease burden following cardiovascular diseases and cancer. Autoimmune diseases have long represented a formidable clinical challenge; currently, there are more than 100 identified types, with approximately 10% of the population affected by one or more of these conditions. Women constitute the most severely impacted demographic, representing roughly 80% of all patients (1, 2). Autoimmune diseases are not precipitated by a single factor but rather emerge from the intricate interplay between genetic susceptibility genes and environmental factors. Ranging from polymorphisms in Human Leukocyte Antigen (HLA) genes to viral infections, chemical exposures, and epigenetic modifications, these multifaceted factors converge into a complex pathogenic network (3). Historically, treatment has predominantly relied on non-specific immunosuppressants; while effective at symptom alleviation, prolonged administration entails various adverse effects, including bone marrow suppression and increased infection risk. Nevertheless, with the emergence of high-dimensional flow cytometry, single-cell sequencing, and spatial transcriptomics, our comprehension of immune cell subsets has reached an unprecedented depth (4). Therapeutic strategies have progressed from TNF-α antagonists to CAR-T cell therapies targeting specific B-cell subpopulations, as the landscape of precision medicine steadily unfolds (5, 6).

Classical immunology posits that B lymphocytes undergo somatic hypermutation and class-switch recombination within germinal center (GC) to differentiate into plasma cells or memory B cells, a process regarded as the indispensable pathway for generating high-affinity antibodies. However, with the advancement of technologies such as single-cell sequencing and flow cytometry, a population of B cells lacking both CD27 and IgD has emerged, termed double-negative (DN) B cells (7, 8). Historically, DN B cells were predominantly characterized as an exhausted or senescent population, particularly in the context of aging and chronic infections. However, recent literature emphasizes that the functional profile of DN B cells is highly context-dependent. While they exhibit exhaustion in age-related immunosenescence, deeper investigation reveals that within the inflammatory microenvironments of autoimmune diseases, DN B cells adopt a hyperactive, pathogenic phenotype. Based on these distinct functional trajectories, they are currently subdivided into four distinct subsets: DN1, DN2, DN3, and DN4. DN1 cells are functionally more akin to conventional memory B cells and potentially derive from GC reactions. In contrast, DN2 B cells exhibit a highly pro-inflammatory phenotype and are believed to originate from extra-GC reactions. Research regarding DN3 and DN4 B cells remains relatively limited at present, though recent studies have begun to elucidate their emergence and potential roles in specific contexts, such as COVID-19 infections and allergic diseases (8–10).

The accumulation of DN B cells, particularly the DN2 B cell subset, in autoimmune diseases was initially identified in patients with systemic lupus erythematosus (SLE), where the proportion of DN2 B cells exhibited a significant positive correlation with disease activity scores (11, 12). Subsequently, the pathogenic roles of DN B cells have been progressively confirmed in other autoimmune disorders, such as rheumatoid arthritis (RA) and Sjögren’s syndrome (13, 14). In patients with autoimmune diseases, there exists a substantial amount of apoptotic cell debris; the impaired clearance of this debris leads to the accumulation of endogenous nucleic acids. These substances provide signals to Toll-like receptors 7 and 9 (TLR7/9) on the B-cell surface, inducing naive B cells to directly enter the extra-GC differentiation pathway (11, 15). However, unlike the rigorous immune selection within GCs, this pathway lacks a negative selection mechanism, enabling autoreactive B cells to survive and rapidly differentiate into antibody-secreting cells (ASCs). These cells produce autoantibodies and pro-inflammatory cytokines in local tissues, leading to the continuous exacerbation of organ damage (16, 17).

Over the past decade, B-cell targeting strategies have been extensively validated in various immune-mediated diseases. Particularly in SLE and RA, B-cell depletion therapies, exemplified by chimeric antigen receptor (CAR) T-cell therapy, have achieved remarkable clinical efficacy, underscoring the pivotal role of the B-cell lineage in disease progression. However, non-specific B-cell depletion possesses inherent limitations, such as the potential compromise of protective immune cell subsets and inter-individual variability in clinical response (4). These challenges have prompted researchers to shift their focus toward more granular B-cell subpopulation research. DN B cells, as a distinct B-cell subset, have demonstrated immense potential in the study of autoimmune diseases. This necessitates a more profound understanding of the development and functional profiles of DN B cells. Elucidating the functions of DN B cells in autoimmune diseases facilitates a deeper revelation of disease mechanisms and their regulation, particularly regarding the advancement of targeted therapeutics. This review aims to summarize the developmental pathways of DN B cells in autoimmune diseases and focus on analyzing their roles in disease progression, with the goal of providing novel perspectives for deciphering disease complexity and exploring innovative therapeutic strategies.

## 2 Classification of double-negative B cells

Upon maturation, B cells can be categorized based on the expression patterns of surface markers IgD and CD27 into: IgD^+^CD27^−^ naïve B cells, IgD^+^CD27^+^ unswitched memory B cells, IgD^−^CD27^+^ switched memory B cells, and IgD^−^CD27^−^ DN B cells. DN B cells account for approximately 5% of total peripheral B cells in healthy individuals and can be further subdivided into four subsets: DN1, DN2, DN3, and DN4. These subpopulations exhibit distinct immunological characteristics under various pathophysiological conditions (7). DN1 B cells represent the predominant component of the DN B-cell pool in healthy individuals and are defined by a CD11c^-^CD21^+^CXCR5^+^ phenotype. DN2 B cells are currently the most thoroughly investigated subset; due to their potent pro-inflammatory properties, they are more active during disease states and are characterized as CD19^bright^CD11c^+^CD21^−^CXCR5^−^. In 2020, researchers further identified the DN3 subset in COVID-19 patients, defined as CD11c^−^CD21^−^CXCR5^−^. DN4 B cells represent a relatively novel and controversial subpopulation, initially identified in studies involving allergic reactions; this subset is enriched for IgE expression and exhibits a CXCR5^+^CD24^+^CD21^+^ phenotype (7, 18, 19). The phenotypic characteristics, developmental origins, primary functions, and associated disease contexts of the four DN B-cell subsets are summarized in **Table 1**.

**Table 1:** Comparative characteristics of double-negative (DN) B-cell subsets.

Subset	Phenotype	Developmental origin	Primary function	Disease context
DN1	CD11c− CD21+ CXCR5+	Extrafollicular pathway	Quiescent; memory-like precursor	Healthy homeostasis (predominant)
DN2	CD11c+ CD21− CXCR5−	Extrafollicular (TLR7/IFN-γ driven)	Pro-inflammatory; autoantibody secretion	Autoimmune diseases (e.g., SLE, RA, MS)
DN3	CD11c− CD21− CXCR5−	Debated (Intermediate vs. distinct)	Tissue-homing; local T-cell interaction	Specific infections (e.g., COVID-19)
DN4	CD24+ CD21+ CXCR5+ IgE+	Debated (Mucosal vs. Th2-driven)	IgE-mediated response; Th2 deviation	Allergic diseases

In addition to the four conventional subsets mentioned above, another model proposed by Szelinski et al., based on the expression levels of CD19 and CXCR5 (20), categorizes DN B cells into three populations: DN^int^, DN^hi^, and DN^low^. Distinct DN B-cell populations also exist within specific infection contexts. For instance, atypical B cells identified in malaria research are also IgD^−^CD27^−^and express CD11c, T-bet, and FCRL5, exhibiting a phenotype highly similar to that of DN2 B cells (21). In the tumor microenvironments of non-small cell lung cancer and nasopharyngeal carcinoma, DN B cells exhibit significant enrichment. Studies have found that the frequency of tumor-infiltrating DN B cells correlates with the immunosuppressive state of the TME (22, 23). During the aging process, a class of DN B cells considered exhausted accumulates in the blood of elderly individuals; chronic immune pressure leads to immunosenescence or functional exhaustion of B cells, subsequently downregulating CD27 expression, which is characterized by telomere shortening, downregulated expression of the pro-apoptotic gene BCL2, and reduced levels of antigen-presenting molecules (24).

## 3 Origin of double-negative B cells

DN1 B cells constitute the largest fraction within the DN B cell pool of healthy individuals (25). These cells express the chemokine receptor CXCR5 and complement receptor CD21 while lacking the activation marker CD11c, presenting a phenotypic profile nearly indistinguishable from classical memory B cells—a similarity further corroborated by single-cell RNA sequencing revealing significant transcriptomic overlap and shared clonal signatures (7). This suggests a common progenitor, from which one subset upregulates CD27 to become classical memory B cells during development, while the other remains in a CD27−DN1 B-cell state. The developmental origin of DN1 B cells has been a subject of ongoing investigation. Recent evidence indicates that DN1 B cells are primarily generated through an extrafollicular route rather than the classical germinal center (GC) reaction. Although bypassing the classical GC, the immunoglobulin genes of DN1 B cells still harbor varying degrees of somatic hypermutation, suggesting that this specific extrafollicular pathway can also support distinct affinity maturation processes (26). Research indicates that DN1 B cells generate long-term immune memory following vaccination, persisting for years. Such durability is a characteristic feature of GC-derived cells, contrasting with the typically shorter lifespan of cells generated via extrafollicular (EF) pathways (27). Single-cell trajectory analysis demonstrates a strong developmental flux from DN1 B cells toward the classical memory population, supporting their role as a “pre-memory” subset (7). Experimental evidence confirms that under specific stimuli, such as CpG, CD27−DN1 B cells can upregulate CD27 expression, thereby fully converting into a classical memory B cells phenotype (28, 29). Emerging evidence suggests that DN1 B cells are not merely a transient state. Studies have identified a unique TP63-associated transcriptional signature and an anti-apoptotic profile in DN1 B cells, enabling their long-term survival post-antigen clearance. Unlike classical memory B cells, DN1 B cells may evolve into a specialized subpopulation capable of re-entering the GC to facilitate further antibody affinity maturation.

DN2 B cells are regarded as precursors to plasma cells. The most prominent hallmark of DN2 B cells is the high expression of the transcription factor T-bet, which promotes B-cell class switching to IgG1 and IgG3 (11, 30). These IgG subclasses possess potent complement-fixing and Fc-receptor-binding capacities; in autoimmune contexts, they serve as the primary drivers of tissue inflammation and immune complex-mediated organ damage (31). Elevated T-bet levels induce the expression of the transcription factor ZEB2, which synergizes with T-bet to enforce a terminal differentiation program, rapidly converting B cells into short-lived plasmablasts (32, 33). Another highly expressed protein in DN2 B cells is FCRL5; in coordination with CD21, this molecule significantly enhances downstream B-cell receptor (BCR) calcium signaling and elevates B-cell activation intensity (34). DN2 B cells also exhibit markedly high IRF4 expression coupled with extremely low IRF8 expression. This reciprocal shift in the IRF4:IRF8 ratio serves as a decisive signal for plasma cell differentiation (35). The progenitors of DN2 B cells are activated naive B cells, and their transformation into the DN2 state depends on specific microenvironmental stimuli (7). Upon the simultaneous receipt of (Interferon) IFN-γ and TLR7 signals in the absence of classical T-cell-derived help, B cells bypass the canonical GC pathway. Instead, they differentiate into DN2 B cells, which are capable of the rapid production of low-mutation, high-affinity autoantibodies (11, 36).

DN3 B cells have been increasingly recognized in recent studies of COVID-19 and fibrotic diseases, characterized as a highly activated “pre-plasmablast” population (10, 37). Activated DN3 B cells also express SLAMF7; the expression of this receptor may be linked to their plasma cell differentiation potential and their interactions with T cells (38). A defining feature of DN3 B cells is their potent tissue tropism. In COVID-19 pulmonary lesions, DN3 B cells are not restricted to the circulation but extensively infiltrate damaged organs (39). Within the tissue microenvironment, DN3 B cells interact with CD4+ cytotoxic T lymphocytes and directly drive local inflammatory infiltration and fibrosis via the CXCL13–CXCR5 chemotactic axis (40). Compared to DN2 B cells, DN3 B cells exhibit transcriptional signatures that more closely mirror those of plasma cells. They express exceedingly high levels of J-chain and PRDM1, accompanied by a significant downregulation of surface CD19 expression (7). As CD19 expression is naturally lost upon entering the terminal differentiation stage of B-cell development, this indicates that DN3 B cells represent a rapid transitional phase toward effector plasma cells.

DN4 B cells represent the most recently identified and currently least characterized subset within the DN B cell family. In contrast to the pro-inflammatory phenotype of DN2 B cells, DN4 B cells exhibit remarkably low T-bet expression. The transcriptomic features of DN4 B cells align more closely with Th2 signaling pathways, suggesting that DN4 B cells are not generated in response to potent Type I inflammatory signals, but rather represent a differentiation lineage tailored for specific Th2 environments or mucosal antigen challenges (7). Single-cell RNA sequencing data clearly demonstrate a profound enrichment of IgE transcripts within the DN4 B-cell subset. This indicates that DN4 B cells may play a pivotal role in allergic diseases such as asthma and atopic dermatitis (41). In these scenarios, DN4 B cells may serve as a reservoir for IgE ASCs, becoming rapidly mobilized upon allergen re-exposure. Additionally, in specific clinical contexts, such as convalescent COVID-19 patients, a modest increase in the proportion of DN4 B cells has also been observed (9, 39).

Rather than functioning as isolated entities, these four subsets represent dynamically distinct biological trajectories with divergent functional roles. Biologically, while DN1 cells serve as relatively quiescent, memory-like precursors capable of long-term survival, DN2 cells act as the hyperactive, pro-inflammatory engine of autoimmunity, rapidly differentiating into autoantibody-secreting cells under TLR7 and IFN stimulation. In contrast to the systemic, Type I inflammatory nature of DN2 cells, the DN3 and DN4 subsets exhibit specialized functional divergence: DN3 cells are uniquely characterized by their robust tissue-homing capacities and direct T-cell interactions within local lesions, whereas DN4 cells completely deviate from this pro-inflammatory axis, showing an exclusive association with Th2-driven and IgE-mediated allergic responses. This functional stratification highlights how the DN B-cell pool is intricately partitioned to address distinct immunological and pathological challenges.

**Figure 1:**
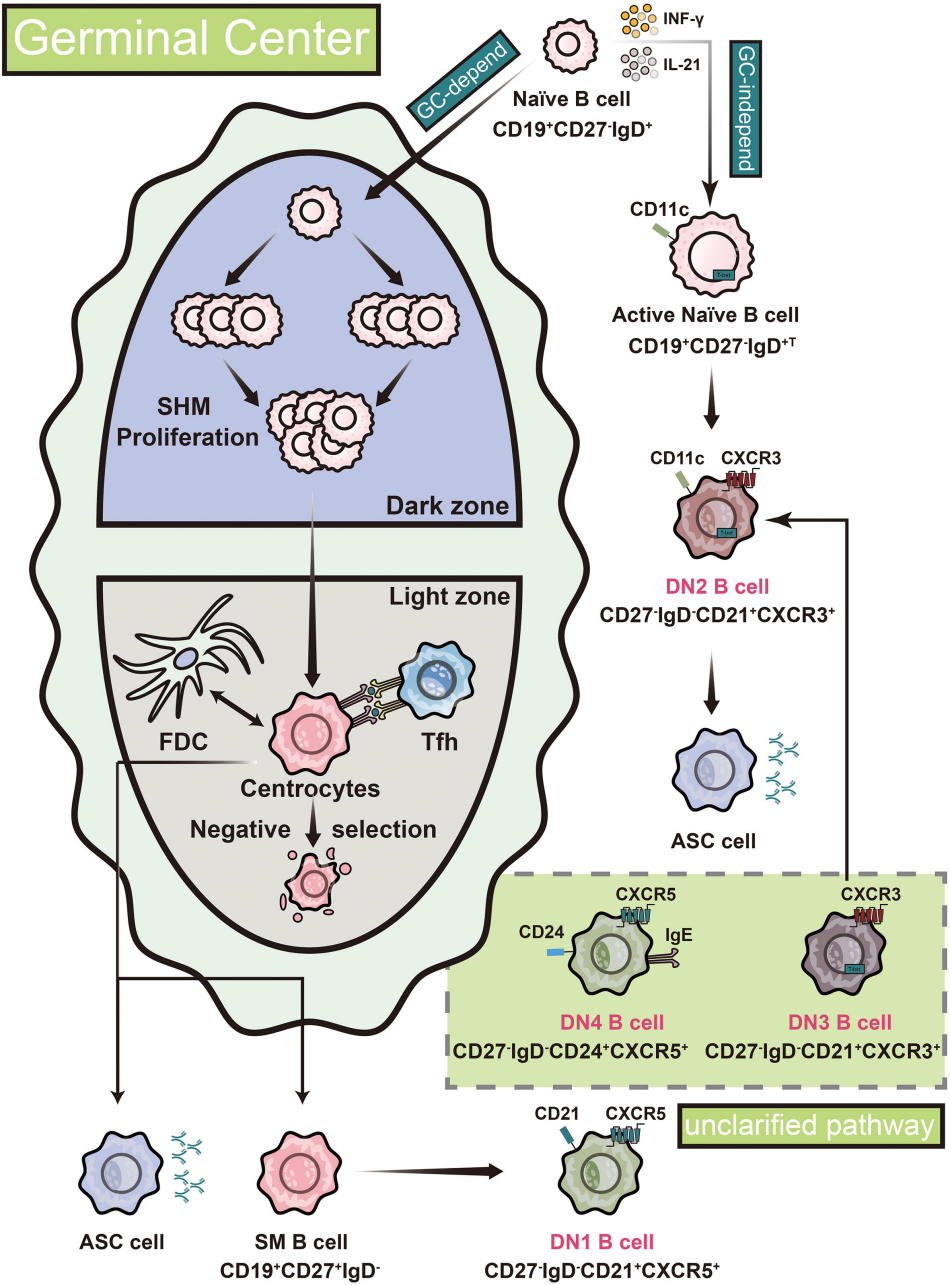
Proposed developmental pathways and phenotypic characteristics of double-negative B-cell subsets. Naïve B cells may enter germinal-center-dependent or extrafollicular differentiation pathways. Germinal-center reactions involve B-cell proliferation, somatic hypermutation, and selection in the dark and light zones, generating antibody-secreting cells, switched memory B cells, and DN1 B cells. Under cytokine and Toll-like receptor stimulation, activated naïve B cells may differentiate through germinal-center-independent pathways into DN2 B cells and antibody-secreting cells. The developmental origins of DN3 and DN4 B cells remain incompletely defined. Representative surface markers and relevant cytokines are indicated.

Despite the proposed developmental trajectories, significant controversies remain regarding the classification and origin of DN B-cell subsets, particularly DN3 and DN4. It is still debated whether DN3 cells represent a distinct, terminally differentiated lineage with specific tissue-homing properties or merely a transient transitional state bridging hyperactive memory cells and extrafollicular plasmablasts. Similarly, the exact origins of DN4 cells are highly controversial; while their IgE enrichment suggests a mucosal or Th2-driven origin, it remains entirely unclear whether they arise from conventional germinal center reactions or specialized extrafollicular pathways. Furthermore, the reliance on varying phenotypic markers and gating strategies across different studies complicates a unified classification system for these rare subsets. These ongoing controversies highlight the urgent need for standardized consensus markers, longitudinal lineage tracing, and high-resolution spatial multi-omics to definitively map their elusive trajectories. The proposed developmental trajectories and phenotypic characteristics of the four DN B-cell subsets are summarized in **Figure 1**.

## 4 Double-negative B cells in autoimmune diseases

### 4.1 Systemic lupus erythematosus

SLE is a multi-organ autoimmune disease characterized by the profuse production of autoantibodies derived from aberrant B-cell activation and differentiation. Within this landscape, DN B cells represent a critical pathogenic subset and a primary source of autoantibody production (11, 20, 42). Research identifies DN2 B cells as the most prominently expanded subset in SLE; their elevated absolute counts and proportions correlate strongly with the incidence of lupus nephritis, 24-hour urinary protein excretion, and high titers of anti-dsDNA and anti-Smith antibodies (11, 12, 43). In studies of African American female patients, DN2 B-cell expansion frequently predicts more severe clinical manifestations and extensive organ involvement. In select cases of young SLE patients presenting with acute flares and severe multi-organ damage, flow cytometric profiles reveal that nearly all non-plasmablast CD19^+^B cells have irreversibly transitioned to a DN2 phenotype (44, 45). Furthermore, recent prospective cohort studies have further revealed a significant expansion of the DN3 B-cell subset in female patients, with its proportion positively correlating with SLEDAI scores (46).

IL-21, a regulator of B-cell activity primarily produced by follicular helper T (Tfh) cells, enhances BCR signaling. Research indicates that IL-21 induces T-bet expression via the STAT3 signaling pathway, driving the transition of activated naive B cells into the DN2 B-cell subset (47). In SLE, Type I and II IFNs are characteristically elevated; specifically, IFN-γ serves as a pivotal signal for T-bet induction and CD11c upregulation, synergizing with TLR7 to ensure B-cell differentiation into the DN2 lineage (48). Additionally, the recently identified type III IFN, IFN-λ, participates in DN2 differentiation by upregulating its receptor, IFNLR1 (49). BAFF further supports B-cell survival and activation, correlating positively with SLE disease activity. Evidence suggests that excessive BAFF weakens the negative selection of autoreactive B cells, permitting their survival and migration to “immunological sanctuaries.” Within these niches, they evade clearance and differentiate into pathogenic populations, including DN B cells (50). In the pathological milieu of SLE, the circulation and tissues are laden with nucleic-acid-rich extracellular traps and RNA-containing immune complexes. Acting as endogenous TLR7 ligands, these substances trigger TLR7 overactivation, which fosters spontaneous GC formation and is closely linked to DN2 expansion (51). Furthermore, while the lncRNA XIST normally mediates X-chromosome inactivation, its dysregulation in SLE-associated DN B cells allows the TLR7 gene to escape silencing. This biallelic TLR7 expression hypersensitizes B cells to self-antigens, driving the direct conversion of naive cells to the DN2 phenotype (52). Conversely, TLR9 appears to counteract TLR7 by dampening inflammatory responses; its deficiency exacerbates TLR7-driven pathology in MRL/lpr mice. Consequently, dual strategies that inhibit TLR7 while augmenting TLR9 activity may offer a therapeutic avenue to control DN B-cell-mediated autoimmunity (53).

The transcription factor T-bet, encoded by TBX21, is a pivotal driver of B-cell differentiation into DN B cells, particularly the DN2 subset. Studies in MRL/lpr mice demonstrate that T-bet promotes the rapid formation of autoantibodies and GCs while enhancing the pro-inflammatory profile of DN2 cells. Accordingly, Tbx21 deficiency significantly reduces autoantibody titers and ameliorates disease symptoms (54). T-bet also coordinates with other factors to downregulate transcriptional programs that typically inhibit effector B-cell differentiation; specifically, the silencing of BACH2 facilitates the downstream progression of DN2 B cells (55). Recent multi-omics analyses further reveal profound metabolic aberrations in these cells. The expansion of DN2 B cells is coupled with a distinct metabolic reprogramming, shifting from relatively quiescent mitochondrial oxidative phosphorylation to highly active aerobic glycolysis (56). This metabolic switch provides the bioenergetic and biosynthetic foundation required for rapid proliferation and robust immunoglobulin synthesis within the hypoxic, inflammatory microenvironments and during target organ infiltration.

Following EF maturation, plasmablasts and long-lived plasma cells derived from DN2B cells emerge as the primary drivers of autoantibody production in SLE (11). These cells secrete high titers of pathogenic autoantibodies that specifically recognize endogenous nucleic acid fragments. Upon binding circulating self-antigens, these antibodies form immune complexes that deposit in organs such as the kidneys, joints, and vasculature, where they activate the complement system and trigger inflammation, further exacerbating tissue damage (57). DN2B cells highly express CXCR3, a receptor that directs cellular migration toward inflamed tissues. The identification of CXCR3^+^B cells within the renal tissues of patients with lupus nephritis suggests that DN2B cells may infiltrate the renal interstitium and glomeruli. Once infiltrating, DN2B cells secrete pro-inflammatory cytokines, intensifying local renal inflammation. Furthermore, DN2B cells are highly sensitive to pro-inflammatory cytokines (43); in the SLE milieu, elevated levels of these factors further drive the rapid differentiation of DN2B cells into ASCs. Characterized by the expression of T-bet and CD11c, DN2B cells possess antigen-presenting and pro-inflammatory capacities, enabling them to interact with circulating Tfh cells (58, 59). The activation of these cTfh subsets correlates positively with DN2 expansion, anti-dsDNA antibody levels, and SLEDAI scores. Finally, DN3 B cells, which are known to infiltrate tissues and contact CD4^+^T cells in other immune disorders, are also hypothesized to drive local inflammation in SLE (46).

Bruton’s tyrosine kinase (BTK) is constitutively activated in DN B cells. In lupus mouse models, BTK inhibition significantly depletes DN B cell populations, ameliorates renal pathological damage, and reduces autoantibody titers (60). JAK-STAT signaling serves as the central driver of DN B cell differentiation; accordingly, the JAK inhibitors tofacitinib or baricitinib limit the accumulation of these cells *in vivo (*61). ZEB2 has been identified as a pivotal transcription factor governing DN B cell formation. Research demonstrates that mice with B-cell-specific ZEB2 deficiency are protected from TLR7-induced lupus symptoms, as the loss of ZEB2 arrests the differentiation of B cells into the DN subset and markedly reduces autoantibody levels (62). In SLE mouse models, high T-bet expression promotes the formation of autoantibodies and GCs; conversely, T-bet inhibition prevents GC development, thereby mitigating renal injury and slowing disease progression (54). Within the immune landscape of SLE, DN B cells exhibit a hypermetabolic profile to meet the energetic demands of rapid differentiation. While they predominantly shift toward aerobic glycolysis, their residual mitochondrial activity and elevated reactive oxygen species (ROS) production remain critical for their survival and pathogenic function. Metformin, a classic metabolic regulator, has demonstrated potential in suppressing DN B cell differentiation in murine models by blocking mitochondrial respiratory chain complex I and reducing mitochondrial reactive oxygen species (ROS) levels (63).

### 4.2 Rheumatoid arthritis

RA is an autoimmune disease characterized by synovitis that can ultimately lead to joint deformity. Studies clearly indicate that the DN2 subset undergoes significant expansion in RA patients (14). This expansion of DN2 B cells shows high clinical relevance and tissue specificity during the course of RA. Reports show that the frequency of DN2 B cells in the peripheral blood of RA patients is significantly higher than in healthy individuals (64, 65). This increase is independent of patient age, suggesting it is driven by autoimmune abnormalities. More pathologically significant is the marked enrichment of DN2 B cells within the synovial tissue of affected joints. Research demonstrates that the proportion of DN2 B cells in synovial tissue is substantially higher than levels in the peripheral blood of the same patient. The expansion of this cell subset correlates positively with the degree of joint space narrowing and cartilage destruction, and is more pronounced in ACPA-positive patients, establishing it as a key immunological indicator for assessing joint damage (14).

Evidence indicates that B cell dysregulation in RA patients is already apparent at the naïve repertoire stage. The number of activated naïve B cells in the blood of RA patients is twice that of healthy controls. These cells have phenotypically lost CD21 and CD24, yet are epigenetically primed to respond to TLR7, serving as direct precursors to DN2 B cells (14). In ACPA-positive RA patients, naïve B cells widely exhibit upregulation of HLA-DR; in a healthy state, HLA-DR expression in naïve B cells is typically maintained at low levels until antigen stimulation occurs. This suggests these cells are in a pathological pre-activated state, ready to participate in pro-inflammatory responses. RA synovial fluid is enriched with damage-associated molecular patterns, such as RNA and DNA fragments released by apoptotic cells. DN2 B cells exhibit hypersensitivity to TLR7 signaling induced by endogenous nucleic acid ligands, providing a constant signal for sustained DN2 cell activation (66). As the core transcription factor of DN2 cells, T-bet expression is strictly regulated by IFN-γ and interleukin-21. Within the RA synovial microenvironment, Th1 and Tfh cells produce high levels of IFN-γ and IL-21, which induce the direct conversion of naïve B cells toward a DN2 phenotype, bypassing the typical GC (66). The SWEF protein family maintains B cell response homeostasis; research shows that SWEF protein deficiency leads to IRF5 dysregulation in response to IL-21, thereby triggering the expansion of DN2 B cells (14, 30).

In RA, DN2 B cells drive disease progression across multiple dimensions through antibody production, antigen presentation, and direct induction of bone destruction. Their primary role in the RA synovium is serving as the foremost source of pathogenic ASCs. RNA trajectory inference and BCR repertoire clonal analysis demonstrate that synovial B cells transition from DN2 to ASCs; during this process, B cell markers such as MS4A1 are progressively downregulated, while key plasma cell markers including JCHAIN, PRDM1, and PPIB rise significantly (67). Because this differentiation occurs in EF regions, the resulting plasma cells secrete ACPA and RF that typically exhibit low mutation levels but high polyreactivity and local pro-inflammatory activity (68). These form abundant immune complexes within the joint cavity, activating the complement system and inducing severe tissue damage. Phenotypically, DN2 B cells express high levels of HLA-DR and the co-stimulatory molecule CD86, granting them potent professional antigen-presenting capabilities. Within the RA synovium, DN2 B cells efficiently capture citrullinated self-antigens and present them to pathogenic CD4^+^T cells. This local T–B cell interaction does not depend on typical lymphoid structures, forming a self-sustaining synovial inflammatory circuit that maintains synovial hypertrophy and the continuous release of pro-inflammatory cytokines (14). Furthermore, DN2 B cells highly express SOX5, a transcription factor that reduces B cell proliferation but enhances their capacity for plasmablast differentiation and antibody production (69). In the synovial fluid of diagnosed RA patients, expanded DN2 B cells specifically express RANKL—the key factor for osteoclast differentiation—thereby directly promoting bone erosion at the joint margins (70, 71).

By targeting CD20, rituximab achieves broad-spectrum depletion of mature B cells, including the DN1 and DN2 subsets. Clinical data confirm that favorable therapeutic responses to rituximab in RA patients are frequently accompanied by a significant reduction in the proportion of T-bet^+^ B cells (72). Long-term administration of tocilizumab (TCZ) markedly reduces the mutation frequency of BCR gene rearrangements in DN B cells. This indicates that IL-6R blockade maintains the B-cell pool at a lower activation state by disrupting the EF pathway and inhibiting the generation of hypermutated memory cells (66). While IgA^+^ plasmablasts play a pivotal role during the mucosal disruption phase preceding RA onset, TCZ significantly reduces the abundance of IgA^+^ DN B cells—a decline that occurs in synchronization with falling serum IgA levels and IgA-type rheumatoid factor (66). JAK inhibitors function by blocking the core signaling pathways that induce T-bet expression; in RA patients, JAK inhibitors treatment significantly downregulates the surface expression of CD40, CD80, and CD95 on DN B cells and reduces AKT phosphorylation following BCR stimulation. By correcting the activity imbalance between SYK and AKT kinases within DN B cells, JAK inhibitors limits premature B-cell activation and survival, thereby promoting clinical remission (73).

### 4.3 primary Sjögren′s syndrome

Primary Sjögren’s Syndrome (pSS) is a complex systemic autoimmune disorder defined by chronic lymphocytic infiltration of the exocrine glands. This infiltration drives a progressive loss of glandular function, manifesting clinically as persistent oral and ocular dryness alongside extensive systemic involvement (74, 75). Multiple flow cytometric studies consistently demonstrate that both the percentage and absolute count of peripheral CD19^+^CD27^−^IgD^−^ DN B cells are significantly reduced in treatment-naive pSS patients compared to healthy controls (13, 76, 77). This depletion is driven by the targeted migration and aberrant recruitment of DN B cells to the salivary and lacrimal glands. High expression of the chemokine receptors CXCR3 and CCR6 allows these cells to track inflammatory ligands, exiting the circulation to infiltrate the exocrine tissues (78). This mechanism is most pronounced in patients with severe glandular involvement and high disease activity, where peripheral DN B cell counts decline further. The IFN signature represents the core transcriptomic hallmark of pSS. More than half of all patients exhibit high levels of IFN activation, a phenotype typically associated with increased disease severity and higher autoantibody titers (79). Within patient strata defined by B-cell-specific IFN−α signatures, the DN2 B cell subset is significantly expanded in IFN−α-positive individuals. Another aberrant population is the CD21^−^ DN B cell (76). These cells expand markedly in pSS patients with lymphoproliferative diseases, and their frequency correlates positively with lymphoma risk. Although CD21 downregulation is traditionally linked to B cell anergy, in pSS, these cells remain highly sensitive to Toll-like receptor stimulation and are enriched with clones secreting autoantibodies, such as rheumatoid factor.

In patients with pSS, elevated IFN−α levels lower cellular activation thresholds, rendering previously dormant autoreactive B cells hypersensitive and driving their EF transformation into DN B cells and plasmablasts (80, 81). This transition, corroborated by Slingshot trajectory analysis, is sustained by the robust expression of IFN-stimulated genes (ISGs) such as STAT1,STAT2, and IFI44. Dysregulation of the IL-7/IL-7R signaling axis represents another pivotal factor driving DN B cell abnormalities. While mature B cells typically lose IL−7R expression under physiological conditions, pSS patients exhibit marked IL−7R upregulation on DN B cells, particularly within the DN2 subset. High levels of IL−7 secreted by salivary gland epithelial cells bind to these receptors to activate the JAK-STAT5 pathway, providing critical proliferative and anti-apoptotic signals that facilitate the persistent survival of these cells within inflamed tissues (76, 82). Moreover, excessive TLR7 activation has been identified as the initiating driver of T−bet^+^ DN B cell expansion. Within the pSS microenvironment, endogenous Ro/SSA-containing complexes function as ligands that induce abnormal B cell differentiation into the DN B phenotype via a TLR7-MyD88-dependent pathway (83). Research further confirms that TLR7 agonist stimulation potentiates higher levels of plasmacytoid differentiation and class-switch recombination in naive B cells from pSS patients, directly leading to a massive expansion of pathogenic DN2 B cells. Transcriptomic profiling reveals distinct metabolic remodeling in DN B cells, characterized by the downregulation of oxidative phosphorylation and translation alongside increased amino acid and lipid utilization—a metabolic shift that meets the high bioenergetic demands of an active autoimmune response (13).

DN B cells actively drive lymphocytic infiltration of the salivary glands through the expression of receptors such as CXCR3. Imaging mass cytometry demonstrates that within heavily infiltrated lesions, DN B cells interact intimately with pathogenic T cells to construct tertiary lymphoid structures and ectopic GCs (84, 85). Beyond evading immune tolerance checkpoints, these cells directly produce high-titer autoantibodies within the glandular tissue; this local accumulation of cytokines and antibodies establishes a deleterious immune microenvironment (86). As inflammation persists, glandular atrophy and ductal loss ensue, with damaged tissue ultimately replaced by fibrous or adipose deposition; meanwhile, secreted pro-inflammatory cytokines further compromise the secretory function of acinar epithelial cells. The DN B cell fraction also mirrors the extent of systemic pSS involvement. A machine-learning-based clustering study of 137 patients identified Cluster A as having the highest DN B cell proportion (32.26%), which corresponded to significantly higher ESSDAI scores compared to other subgroups (p=0.034). Depletion of peripheral DN B cells correlates significantly with neutropenia, lymphadenopathy, and cutaneous involvement. While pSS patients carry a risk of non-Hodgkin lymphoma manifold higher than the general population, the DN B cell compartment serves as a critical nexus for malignant transformation. In Cluster A—characterized by high DN B cell frequencies—the incidence of lymphoma reaches 20%, whereas Clusters C and D, with lower DN frequencies, show 0% incidence (84). This divergence likely arises from the monoclonal expansion of autoreactive CD21^−^ DN B cells; under the sustained pressure of chronic autoantigens and TLR signaling, these cells eventually undergo malignant evolution into low-grade B-cell lymphomas, driven by chromosomal abnormalities.

Studies have confirmed that pathogenic DN B cells in patients with pSS highly express IL-7R, identifying IL-7R-targeted therapy as a promising strategy. Anti-IL-7R antibodies competitively bind the receptor, thereby inhibiting the activation of the downstream STAT5 signaling pathway. Consequently, IL-7R-high DN B cells lose anti-apoptotic protein expression and undergo programmed cell death, reducing the pathogenic DN B cell population *in vivo (*82). The IFN signature is considered the core molecular hallmark of Sjögren’s syndrome. IFN induces the differentiation of B cells into DN B cells by upregulating the expression of TLR7, TLR9, and the chemokine receptor CXCR3. Targeting the IFN signaling pathway to block downstream STAT1 activation therefore represents a potential therapeutic strategy for pSS. Notably, anifrolumab—an antagonist targeting the IFN receptor subunit 1—has demonstrated significant efficacy in SLE (13, 87).

### 4.4 Multiple sclerosis

Multiple Sclerosis (MS) is an autoimmune disorder primarily defined by inflammatory demyelination of the central nervous system white matter. It is characterized by an immune-mediated assault on the myelin sheath, resulting in the hallmark dissemination of lesions in both time and space (88). As the field of neuroimmunology has evolved, the pathological paradigm of MS has shifted from a purely T-cell-mediated model toward a complex B-T cell synergistic network (89). Beyond driving CNS injury through antibody production, B cells serve as pivotal antigen-presenting cells and secretors of pro-inflammatory cytokines, thereby fueling the chronic progression of the disease (90). In one study, the DN2 subset (phenotypically characterized as CD21^-^CD11c^+^ DN B cells) was significantly expanded in approximately 22% of young patients with MS. During the process of autoproliferation in MS patients, T−bet+CXCR3+DN B cells are markedly enriched in those who are autoproliferation-positive (91). The molecular signature of these cells is defined by the upregulation of T−bet and various ISGs. These cells undergo clonal evolution, transitioning from a memory state into plasmablasts, a process accompanied by frequent somatic hypermutation. Critically, this subset is densely sequestered within highly inflamed meningeal tissues and ectopic lymphoid structures, where their abundance correlates positively with cerebrospinal fluid cell counts and the severity of spinal cord lesions (89, 92).

DN B cells are also characterized as “aged” memory B cells. In MS patients, chronic antigenic stimulation triggers immune remodeling, whereby memory B cells downregulate CD27 and CD21 while upregulating CD11c and the transcription factor T−bet, transitioning into a DN phenotype—a phenomenon known as premature immunosenescence (91, 93). Given that Epstein-Barr virus (EBV) infection is a requisite precursor for MS, the expression profile of expanded DN B cells in these patients closely recapitulates the phenotype induced by EBV *in vitro*. Consequently, EBV may drive the persistent generation of DN B cells by inducing autoreactive B-cell clones via its latent protein, EBNA1 (94, 95). The primary genetic risk factor for MS, HLA−DR15, appears to be a critical determinant of these DN B-cell dynamics. In MS patients, memory B cells present self-peptides via HLA−DR15 to induce the proliferation of B and T cells in the absence of exogenous antigens, directly precipitating the expansion of the T−bet^+^CXCR3^+^ DN B-cell subset (89, 96).

In patients with MS, DN B cells and CD4^+^Th1 cells aggregate into inflammatory clusters that mimic the GC reactions characteristic of secondary lymphoid organs (89). Serving as primary antigen-presenting cells, B cells leverage costimulatory molecules—such as CD80 and CD86—to activate pathogenic T cells with brain-homing capabilities. Through the secretion of IFN-γ and its downstream signaling, DN B cells not only augment and sustain cognate Th1 responses but also induce pSTAT1 activation in both themselves and surrounding cells, driving more severe central nervous system (CNS) infiltration (97, 98). Concurrently, DN B cells in early MS significantly upregulate the expression of various inflammatory cytokines, including CXCL8, IL-8, IL-18, and VEGFA, directly compromising blood-brain barrier integrity and exacerbating neuroinflammation (98, 99). Facilitated by the surface expression of the CXCR3 chemokine receptor, DN B cells possess the capacity to enter the CNS. These cells traffic to the meninges and spinal cord lesions to participate in the construction of ectopic meningeal lymphoid tissues (92); here, B cells undergo somatic hypermutation and class-switch recombination, differentiating into plasmablasts that produce pathogenic antibodies *in situ*, which ultimately manifest as oligoclonal bands in the cerebrospinal fluid.

Anti-CD20 monoclonal antibodies represent one of the most effective therapeutic strategies for MS. As DN B cells express the CD20 marker, B-cell depletion therapy extensively eliminates these pathogenic cells and their precursors from the peripheral blood, thereby disrupting peripheral pro-inflammatory cascades and curtailing infiltration into the CNS. However, because long-lived plasma cells typically lack CD20 expression, these therapies often fail to fully eradicate oligoclonal bands within the cerebrospinal fluid (98). BTK is a pivotal mediator of BCR signaling. Evidence indicates that BTK inhibitors significantly suppress autoproliferation and the formation of B-T cell clusters in MS. In experimental settings, BTK inhibitors treatment markedly attenuates B-cell activation markers and limits their differentiation into T-bet^+^ pro-inflammatory phenotypes (100). Natalizumab blocks integrin-mediated migration, preventing lymphocytes—including DN B cells—from crossing the blood-brain barrier, which significantly reduces B-cell density in the CSF (101).

### 4.5 Other autoimmune diseases

Increasing evidence implicates DN B cells in the pathogenesis and progression of various autoimmune diseases. In juvenile idiopathic arthritis (JIA), peripheral DN2 B-cell expansion correlates with T-cell activation, suggesting antibody-independent immunomodulatory functions (102). Uveitis represents one of the most severe complications of JIA; patients with ocular involvement exhibit significantly higher proportions of peripheral DN1 B cells than those without (103). In autoimmune pancreatitis, infiltrating macrophages recruit DN cells via the CXCL9-CXCR3 axis, leading to specific DN2 B-cell enrichment in the pancreas (104). DN2 cells are expanded in the peripheral blood and affected tissues of patients with IgG4-related disease (IgG4-RD)—a phenomenon validated in the lungs of IgG4-RD mouse models—suggesting a critical role in immune-mediated injury (105). Research in idiopathic inflammatory myopathy (IIM) has identified an expansion of peripheral DN2 B cells enriched for IFN−λ signaling, suggesting the IFN−λ/DN2 axis may drive pathogenesis and serve as a biomarker for disease activity (106). In inflammatory bowel disease (IBD), peripheral DN B-cell proportions are significantly reduced compared to healthy controls, while their presence in gut-associated lymphoid tissue is markedly increased. This distribution suggests that peripheral DN B cells migrate to inflammatory sites, where they may exacerbate local inflammation by responding to gut microbiota (107). Peripheral DN B cells in axial spondyloarthritis (axSpA) highly express the chemokine receptor CXCR3, highlighting their migratory potential. The proportion of this subset correlates positively with the erythrocyte sedimentation rate (ESR), supporting the role of DN B cells in axSpA progression through the mediation of inflammatory responses (108). Similar expansions of the DN B-cell population are observed in Graves’ disease, Hashimoto’s thyroiditis, and myasthenia gravis (25, 109–111). The predominant DN B-cell subsets, major driving signals, and tissue tropism across different autoimmune diseases are summarized in **Table 2**.

**Table 2:** Horizontal comparative analysis of pathogenic DN B cell subsets.

Disease	Predominant expanded subset(s)	Primary driving Signals	Tissue tropism & infiltration site
SLE	DN2, DN3	IFN-γ, IFN-λ, TLR7, BAFF, IL-21	Renal interstitium and glomeruli
RA	DN2	TLR7, IFN-γ, IL-21 (Th1/Tfh derived)	Synovium
Pss	DN2, CD21- DN B cells	IFN-α, IL-7/IL-7R axis, TLR7	Salivary and lacrimal glands
MS	T-bet+CXCR3+ DN B cells	HLA-DR15-restricted antigen presentation, EBV (EBNA1)	Meninges and central nervous system (CNS) lesions
JIA	DN2 (peripheral), DN1 (uveitis)	T-cell activation (correlates)	Joints, Ocular involvement (uveitis)
AIP	DN2	CXCL9-CXCR3 axis	Pancreas
IgG4-RD	DN2	–	Affected tissues (validated in lungs)
IIM	DN2	IFN-λ signaling	Peripheral expansion
IBD	DN B cells	Gut microbiota response	Gut-associated lymphoid tissue (GALT)
axSpA	CXCR3+ DN B cells	–	High migratory potential

## 5 Translational therapeutic strategies and limitations

While targeting DN B cells holds immense clinical promise, translating these biological findings into precision therapies remains challenging. Current conventional therapies, such as CD20-targeted depletion (e.g., rituximab), effectively eliminate circulating B cells but present significant clinical limitations. They are often hampered by their inability to efficiently eradicate tissue-resident DN B cells sequestered within tertiary lymphoid structures in affected organs, while simultaneously subjecting patients to prolonged immunosuppression and elevated infection risks. Furthermore, BAFF-targeted therapies (e.g., belimumab), though clinically successful in conditions like SLE, may exhibit limited efficacy against specific pathogenic DN B-cell subsets. This limitation arises because the generation of DN2 cells is predominantly driven by BAFF-independent, TLR7/IFN-γ-mediated extrafollicular pathways.

Consequently, emerging translational strategies are shifting from pan-B-cell depletion toward precision interventions. Targeting the unique metabolic vulnerabilities of DN B cells—such as utilizing metformin to inhibit their hyperactive ROS production and glycolytic shifts—offers a promising avenue to specifically curtail pathogenic differentiation without causing systemic B-cell aplasia. Furthermore, precision targeting of specific signaling cascades has shown considerable potential. For instance, BTK inhibitors and JAK inhibitors are being actively investigated to disrupt the hyperactive BCR and cytokine-driven (e.g., IFN-γ) signaling pathways that fuel DN2 differentiation. Similarly, novel biologicals like anti-IL-7R antibodies are emerging as potential tools to disrupt the critical T-B cell crosstalk in the extrafollicular niche. Additionally, developing novel agents that block specific extrafollicular signaling hubs (such as TLR7/9 inhibitors) represents the future of targeted therapy. However, the true clinical applicability of these emerging strategies remains contingent upon overcoming off-target metabolic effects, mapping the precise temporal window for intervention, and rigorously verifying their long-term safety profiles in robust clinical cohorts.

## 6 Conclusions and perspective

In conclusion, DN B cells have transitioned from being initially regarded as exhausted subpopulations to being recognized as pivotal pathogenic drivers in autoimmune diseases. Subsets such as DN2 B cells exemplify the functional remodeling of B cells under chronic inflammatory stress: by bypassing the stringent negative selection of the GC, they establish EF response mechanisms independent of classical immune surveillance, serving as primary sources of pathogenic autoantibodies and pro-inflammatory cytokines. Beyond acting as biomarkers for peripheral disease activity, DN B cells infiltrate target organs via robust migratory capacity, where they synergize with T cells to shape local microenvironments and tertiary lymphoid structures, thereby exacerbating tissue damage.

While the pathogenic landscape of DN B cells is taking shape, several critical gaps remain. First, the unique tissue tropism of DN3 B cells in organ lesions and the potential association of DN4 cells with IgE responses suggest that these subsets play distinct roles from DN2 cells in specific pathological contexts. Elucidating the origins, differentiation trajectories, and *in situ* niches of these rare subsets—leveraging spatial transcriptomics and lineage tracing—will be essential to advancing current knowledge. Second, although TLR7/9, IFN-γ, and IL-21 signaling are known to drive DN2 differentiation, the fundamental decision-making mechanisms that direct B cells toward either high-affinity selection in GCs or rapid EF pathways remain to be clarified. Finally, while current B-cell depletion therapies are effective, they lack specificity. Conventional non-specific depletion eliminates pathogenic populations but simultaneously compromises protective B-cell subsets, increasing the risk of infection. Consequently, future research must prioritize the identification of DN B-cell-specific molecular targets to enable more precise therapeutic interventions.
